# Targeting allosteric regulatory modules in oncoproteins: “Drugging the Undruggable”

**DOI:** 10.18632/oncotarget.354

**Published:** 2011-11-12

**Authors:** Oliver Hantschel, Florian Grebien, Giulio Superti-Furga

**Affiliations:** ^1^ Center for Molecular Medicine of the Austrian Academy of Sciences, Lazarettgasse 14, AKH BT 25.3, 1090 Vienna, Austria; ^2^ Ecole polytechnique fédérale de Lausanne (EPFL), Swiss Institute for Experimental Cancer Research (ISREC), Lausanne, Switzerland

Kinases are often aberrantly activated in various tumor types by overexpression, point mutations, chromosomal translocations and other mechanisms. Therefore, small molecule inhibitors that block the enzymatic activity of kinases have been developed for different targets and are successfully used for the treatment of cancer patients. A major drawback of these molecularly targeted therapies is the frequent development of therapy resistance, often caused by point mutations in the target protein ([1]). This can manifest in short-lived remissions and disease progression. A valuable strategy to counteract these developments is the identification and subsequent targeting of additional sites on the oncogenic driver kinase. To qualify as an allosteric drug target, these regulatory sites need to be essential for the oncogenic activity of the respective kinase. Alternatively, they may represent allosteric attenuators of enzymatic activity, critical protein-protein interactions or downstream signaling pathways. In the past years, several such sites were identified in hallmark kinases. For example, it was shown that the activation of the epidermal growth factor receptor (EGFR) results from the formation of an asymmetric dimer in which the C-terminal lobe of one kinase domain binds to the N-terminal lobe of the other kinase domain and allosterically activates it. The site of interaction and mechanism of kinase activation is reminiscent to that of cyclins in activated cyclin-dependent kinase/cyclin complexes [2]. Also, the activation of the BRAF kinase, which is frequently mutated in tumors depends on the formation of side-to-side homo- and hetero-dimers with CRAF or the pseudo-kinase kinase suppresor of Ras (KSR) [3]. Paradoxically, inhibition of BRAF kinase activity by mutation or certain BRAF kinase inhibitors resulted in activation of the MEK-ERK pathway [3].

The hallmark of chronic myeloid leukemia (CML) is the expression of the oncogenic kinase Bcr-Abl, which arises from the Philadelphia chromosome translocation [4]. Bcr-Abl's high activity is targeted by the ATP-competitive tyrosine kinase inhibitor Gleevec leading to durable remissions in CML patients [4].

Recently, the myristate binding pocket in the C-terminal lobe of the Abl kinase domain was identified as a site for allosteric regulation of Bcr-Abl activity [5]. A small molecule that was engineered to bind to the myristate binding pocket was shown to inhibit Bcr-Abl allosterically [6].

In addition, Bcr-Abl as well as its proto-oncogenic constituent c-Abl (ABL1) have a Src homology 2 (SH2) domain N-terminal to their tyrosine kinase domains - a conserved feature among the class of cytoplasmic tyrosine kinases. The analysis of active Abl conformations lead to the finding that SH2 domains are positive allosteric effectors in cytoplasmic tyrosine kinases via the formation of an intramolecular interface of the SH2 domain with the kinase domain [7]. The SH2-kinase domain interaction in Bcr-Abl was both necessary and sufficient for high catalytic activity of the enzyme. Disruption of this interface led to inhibition of downstream events critical for CML signaling and, importantly, completely abolished leukemia formation in mice [8]. Furthermore, disruption of the SH2-kinase interface increased sensitivity of imatinib-resistant Bcr-Abl mutants to TKI inhibition. To target this interaction, an engineered Abl SH2 binding fibronectin type III-monobody was developed that inhibited Bcr-Abl kinase activity inducing apoptosis of primary CML cells. This validated the SH2-kinase interface as an allosteric target for therapeutic intervention [8].

The exquisite affinity and specificity of monobodies exemplifies its general utility as target validation tools for preclinical studies. On the other hand, the necessity for intracellular delivery that may only be achieved using lentiviral gene transfer or fusion of the monobody to membrane-permeable peptides will likely limit or preclude the use of monobodies as drug-like molecules in clinical applications.

In contrast, small-molecule protein-protein interaction inhibitors have been developed for a number of protein targets, breaking with the dogma that protein-protein interfaces are ‘undruggable’. However, interfering with intramolecular domain interactions (like the SH2-kinase interface in Bcr-Abl) might require very high local concentrations of inhibitor that may be difficult to achieve using intracellular delivery of proteins or peptides. Therefore, we believe that the development of a small-molecule inhibitor of the SH2-kinase domain interface that may be used in combination with approved Bcr-Abl tyrosine kinase inhibitors could be feasible.
